# Cardiovascular Manifestations in Hyperthyroidism: A Cross-Sectional Study in a Tertiary Care Hospital in South India

**DOI:** 10.7759/cureus.25232

**Published:** 2022-05-23

**Authors:** Lakshmi Nijith, Rajesh Ranjan

**Affiliations:** 1 Internal Medicine and Geriatrics, Ahalia Diabetes Hospital, Kozhippara, IND; 2 Community Medicine, Noida International Institute of Medical Sciences, Noida, IND

**Keywords:** tachycardia, echocardiography, ecg, hyperthyroidism, cardiovascular

## Abstract

Background

The involvement of the heart in hyperthyroidism patients has a considerable prognostic value and causes significant morbidity and mortality. However, very little research, particularly among the Indian population, has addressed the most critical cardiovascular symptoms of hyperthyroidism, so this study aimed to assess the cardiovascular manifestations of hyperthyroidism.

Method

The current cross-sectional investigation involved 140 newly diagnosed and untreated confirmed cases of hyperthyroidism of any etiology for nine months. A structured data collection schedule was used to collect patient-specific and pertinent information during OPD hours or after admission. T4, T3, and TSH were measured in 10 mL of blood from each patient. The information was entered into a Microsoft Excel spreadsheet. All tests were carried out with a 5% level of significance.

Results

The mean age of study subjects was 43.2 years. Females made up 85.0% of the subjects in the current study, while males made up 15.0%. The etiology of hyperthyroidism was primarily due to Grave’s disease (59.3%). Heat intolerance (67.9%) was the most typical presenting symptom among the patients. The most common cardiac symptom was palpitation among 76.4% of subjects in the present study. Upon clinical examination, 80.7% of subjects had tachycardia. The ECG showed atrial fibrillation (AF) in 17.9% of subjects. The echocardiogram (ECHO) findings revealed systolic dysfunction in 17.8% of subjects.

Conclusion

Since cardiovascular manifestations are common in patients with thyroid disease and may be the only manifestation of thyroid disease, it is suggested that all patients with thyroid disorders be checked for cardiovascular manifestations. In addition, thyroid function tests should be performed in all patients with unexplained cardiovascular disease.

## Introduction

After diabetes mellitus, thyroid diseases are the most frequent endocrinological illnesses seen in clinical practice. Hyperthyroidism is a condition in which the thyroid gland functions excessively. Thyrotoxicosis is a condition in which the thyroid hormone is produced in large quantities and causes symptoms and signs in the cardiovascular, neurological, ophthalmological, dermatological, GI, and endocrinological systems. The majority of cases of thyrotoxicosis are characterized by hyperthyroidism caused by Grave's disease, multinodular goiter, and toxic adenomas [1]. Thyrotoxicosis is caused by Grave's disease in 60-80% of cases. Thyroid hormone has a wide range of physiological and biochemical effects on the functions of multiple organ systems. The clinical presentation is determined by the duration, severity of the disease, and hormone levels in the blood [2].

Hyperthyroidism manifests itself in various ways, including signs and symptoms such as hyperactivity, heat intolerance, fatigability, weight loss, and neurological, cardiac, dermatological, ophthalmological, GI, and endocrinological manifestations [3]. The T3 and T4 have a large influence on cardiovascular function and produce significant disturbances in hemodynamic function. Therefore, the heart is the primary organ being impacted. Hyperthyroidism is related to tachycardia, increased left ventricle (LV) workload, and supraventricular tachycardia (SVT), such as atrial fibrillation (AF). Tachycardia, wide pulse pressure, AF, rapid pulses, loud first heart sound, and hyperdynamic cardiac apex are major signs of hyperthyroidism [4,5].

The involvement of the heart in hyperthyroidism patients has a considerable prognostic value and causes significant morbidity and mortality. Even more so, with early diagnosis and adequate therapy, affected patients may be able to reverse their condition. AF can lead to secondary problems such as cerebral stroke [6,7,8]. When hyperthyroidism is treated with anti-thyroid medicines, and thyroid hormone levels return to normal, cardiac issues begin to dissipate. The majority of hyperthyroidism problems are prevented by early and rapid detection of cardiac symptoms and signs and early and suitable therapy [9].

Furthermore, the majority of the cardiac features include hyperthyroidism and thyrotoxicosis, presenting symptoms and signs. Therefore, the early detection of heart symptoms is also not a big challenge. In such cases, a detailed clinical examination along with ECG and echocardiogram (ECHO) is required for the early diagnosis of cardiac problems [10,11,12].

The clinical presentation and laboratory features of hyperthyroidism have been the subjects of much research. However, very little research, particularly among the Indian population, has addressed the most critical cardiovascular symptoms of hyperthyroidism. Considering that early diagnosis of hyperthyroidism and early identification of associated complications require knowledge of the cardiac manifestations of hyperthyroidism, this study was aimed at assessing the cardiovascular manifestations of hyperthyroidism.

## Materials and methods

Study setting and design

After receiving ethical approval from the Institutional Ethics Committee (IEC/IRB No. AIHFEC/04/023; Ahalia International Foundation Ethics Committee, Kozhippara), the current clinical descriptive cross-sectional study was conducted for nine months (May 2021 to January 2022) in the Department of Internal Medicine of a tertiary care hospital in Kerala, India.

Study subjects and sample size

Patients (18 years or older) with newly diagnosed and untreated confirmed cases of hyperthyroidism (overt or subclinical) of any etiology who visited the OPD or were admitted to the wards were included in the study. The minimal sample size was determined to be 97, based on a 50% prevalence of cardiovascular manifestations in hyperthyroidism (no specific studies were found in Kozhippara) and a 10% absolute precision. Prior to enrolling subjects in the study, written informed consent was obtained from either the patient or relatives after a detailed explanation of the study's purpose. A consecutive sampling method was used to enroll the study subjects, resulting in a total of 140 patients being enrolled in the study throughout the study's defined duration.

Data and sample collection

Clinical history was gathered during OPD hours or after admission, and patient-specific and pertinent information was acquired through interviews in a structured data collection schedule. Each patient had 10 mL of blood drawn for laboratory tests like complete blood count (CBC), renal function test (RFT), liver function test (LFT), serum electrolytes, fasting lipid profile (serum triglycerides, low-density lipoprotein [LDL], high-density lipoprotein [HDL], total cholesterol), and T4, T3, and TSH (done by radio-immunoassay method). The patient was diagnosed with hyperthyroidism if the serum T3 level was >200 ng/dl, serum T4 level was >12 mcg/dl, or serum TSH level was < 0.3 mU/ml. Also, for each patient, an X-ray chest PA view, ECG, and 2D ECHO were performed to analyze the presence of any cardiac manifestations. In addition, fine needle aspiration cytology (FNAC) thyroid gland and USG neck were performed to confirm the etiology of hyperthyroidism.

Statistical analysis

The data was entered into an MS Excel spreadsheet and analyzed with the SPSS version 26 software. Their baseline demographic, clinical, and laboratory information was used to analyze the results for each group of research patients. Continuous variables were reported as mean SD, whereas categorical variables were presented as numbers and percentages (%). The Kolmogorov-Smirnov test was used to determine whether the data was normal. The non-parametric test was employed if normality was refused. All tests were done at a 5% significance level; an association was considered significant if the p-value was <0.05.

## Results

The mean age of study subjects was 43.2±13.7 years. 42.1% of subjects belonged to the 20-40 year age group, followed by 40.7% belonging to the 41-59 year age group. Females made up 85.0% of the subjects in the current study, while males made up 15.0%. The etiology of hyperthyroidism was primarily due to Grave's disease (59.3%) and multinodular goiter (36.4%). Heat intolerance (67.9%), fatigue (65.0%), and weight loss (49.3%) were the most typical presenting non-cardiac symptoms of the patients, and the majority of the patients (66.4%) had less than one year of duration for such symptoms (Table 1).

**Table 1:** Baseline characteristics of study subjects (N = 140). *Multiple responses

Variables	Number	%
Age group (in years)		
<20	5	3.6
20-40	59	42.1
41-59	57	40.7
>60	19	13.6
Gender		
Male	119	85.0
Female	21	15.0
Etiology of hyperthyroidism (Ultrasonography)		
Grave’s disease	83	59.3
Multinodular goiter	51	36.4
Solitary nodule	6	4.3
Non cardiac Symptoms*		
Heat intolerance	95	67.9
Fatigue	91	65.0
Weight loss	69	49.3
Increased appetite	43	30.7
Diarrhea	23	16.4
Tremor	17	12.1
Duration of symptoms		
<1 year	93	66.4
1-2 years	23	16.4
>2 years	24	17.2
Neurological Signs*		
Tremor	71	50.7
Hyperreflexia	37	26.4
Proximal myopathy	5	3.6
Periodic paralysis	3	2.1

In the present study, more than half of the subjects (56.4%) had T3 levels in the range of 200-399 ng/dl. Almost two-fifths of the subjects (45.0%) had T4 levels in the range of 18-23 mcg/dl. In addition, 50.0% of patients had TSH levels in the range of 0.01-0.009 mU/ml. The FNAC results showed that lymphocytic infiltrates (45.0%) and colloid nodules (40.7%) were featured in more than two-fifths of the subjects (Table 2).

**Table 2:** Laboratory parameters and FNAC findings among study subjects (N = 140). FNAC: Fine needle aspiration cytology.

Parameters	Number	%
T3 level (60-200 ng/dl)		
<200	5	3.6
200-399	79	56.4
400-599	41	29.3
600-799	13	9.3
>800	2	1.4
T4 level (4.5-12.0 mcg/dl)		
12-17	57	40.7
18-23	63	45.0
24-30	20	14.3
TSH level (0.3-5.03 mU/ml)		
<0.009	51	36.4
0.01-0.009	70	50.0
>0.1	19	13.6
FNAC findings		
Lymphocytic infiltrates	63	45.0
Colloid nodule	57	40.7
Follicular cells	7	5.0
Follicular adenoma	8	5.7
Benign nodular goiter	5	3.6

In the present study, the most common cardiac symptom was palpitation among 76.4% of subjects. Also, nearly 20.0% of subjects presented with no cardiac symptoms. Upon clinical examination, 80.7% of subjects had tachycardia, 49.3% had hypertension, and 36.4% had wide pulse pressure. The mean pulse pressure among subjects was 58.3±10.5 mm Hg, and the mean heart rate was 105.8±13.1 per minute. Auscultatory findings showed loud S1 in 27.9% of patients and a systolic ejection murmur (pulmonary area) in 17.1% of subjects (Table 3).

**Table 3:** Cardiovascular signs and symptoms among study subjects (N = 140). *Multiple responses

Variables	Number	%
Symptoms*		
Palpitation	107	76.4
Edema	23	16.4
No cardiac symptoms	28	20.0
Cardiovascular signs*		
Tachycardia	113	80.7
Hypertension	69	49.3
Wide pulse pressure (> 60 mm Hg)	51	36.4
Loud S1	39	27.9
Ejection systolic murmur (Pulmonary area)	24	17.1
Cardiac Failure	20	14.3
Pan-systolic murmur (mitral)	15	10.7
Early diastolic murmur (Aortic area)	9	6.4

The ECG showed sinus tachycardia among 80.7% of subjects and AF among 17.9% of subjects. The normal ECG was noticed in 15.0% of subjects. The chest X-ray showed cardiomegaly among 20.7% of subjects and pulmonary hypertension in 5.7% of subjects. The mean ejection fraction was 63.3±8.7% among study subjects. The ECHO findings revealed systolic dysfunction in 17.8% of subjects, mitral regurgitation in 14.3% of subjects, and left ventricular hypertrophy in 12.1% of subjects. Also, 27.1% of subjects showed no abnormality in ECHO (Table 4).

**Table 4:** ECG, ECHO, and X-ray findings among study subjects (N = 140). *Multiple responses

Variables	Number	%
ECG abnormality*		
Sinus tachycardia	113	80.7
Atrial fibrillation	25	17.9
Left ventricular hypertrophy	18	12.9
Normal	21	15.0
Chest X-ray*		
Normal	111	79.3
Cardiomegaly	29	20.7
Pulmonary hypertension	8	5.7
Echocardiographic abnormality		
Systolic dysfunction	25	17.8
Diastolic dysfunction	15	10.8
Mitral regurgitation	20	14.3
Left ventricular hypertrophy	17	12.1
Pulmonary hypertension	9	6.4
Aortic regurgitation	5	3.6
Tricuspid regurgitation	7	5.0
Mitral valve prolapse	4	2.9
No abnormality	38	27.1

## Discussion

Thyroid hormone affects the heart and cardiovascular system through various direct and indirect pathways. Thyroid hormones alter myocytes by upregulating the alpha (α)-chain while downregulating the beta (β)-chain. It may also have an effect on the sarco/endoplasmic reticulum, increasing calcium uptake during diastole [13-15]. Thyroid hormone also affects myocardial and vascular properties by acting directly on ion channels such as Na/K-ATPase, Na/Ca++ exchanger, and some voltage-gated K channels. Thyroid hormone affects the hemodynamic balance in the body by direct effects on the heart and blood vessels, in addition to its cellular effects. Thyroid hormones may stimulate the body to use oxygen more quickly, enhance metabolic product output, and relax arterial smooth muscle, all of which can lead to peripheral vasodilation [16,17].

In the present study, the mean age of the subjects was 43.2 years, and 42.1% of the subjects belonged to the 20-40 year age group, followed by 40.7% belonging to the 41-59 year age group. This is consistent with the existing literature, which shows that hyperthyroidism is widespread in those aged 20 to 50 years [18]. In the current study, more females than males were affected. This is in line with the existing literature [18]. The most prevalent presenting symptoms in this study were heat intolerance (67.9%), fatigue (65.0%), and weight loss (49.3%). Similarly, in a study by Rajput R et al., the most prevalent presenting symptoms were heat intolerance (82.1%) and weight loss (76.8%) [19]. The most common cause of hyperthyroidism in our analysis was Grave's disease. Grave's disease was responsible for hyperthyroidism in 59.3% of the study subjects. This is slightly lower than the 60-80% reported occurrence [18].

Cardiac symptoms found in hyperthyroidism could be caused by increased sympathoadrenal activity or thyroid hormones directly affecting the heart [20]. Palpitation (76.4%) was the most prevalent cardiac symptom in this study. Palpitation was the most common cardiovascular manifestation in a study by Kandan V et al. (78%), followed by dyspnea/breathlessness (26%) and chest pain (4%) [21]. Tachycardia (pulse rate >100 beats per minute) was observed in 80.7% of the participants in this study, which was comparable to Kandan V et al., and Zargar AH et al. studies [21,22].

AF was seen in 17.9% of the patients in the present study, which was comparable to the study by Kandan V et al., which found a 28% incidence rate of AF [21]. However, the proportion in our study was considerably higher than that reported by Zargar AH et al., which found a prevalence of 8.9% [22]. The presence of a low TSH level in the blood is an independent risk factor for the development of AF [23]. In patients with hyperthyroidism, AF has been shown to cause higher mortality and morbidity due to embolic events [24,25].

P-maximum and P-wave dispersion are key ECG findings for identifying paroxysmal AF [20]. Systolic dysfunction was observed in 17.8% of the patients in this study, similar to the study done by Mercé J et al., which revealed systolic dysfunction among 18% of subjects [26]. However, in their research, Kandan V et al. found a significantly lower prevalence of systolic dysfunction (3%) [21].

Limitations

This is the first local research that we are aware of identifying cardiovascular manifestations in hyperthyroid individuals. However, the research is not without limitations. The sample size was limited and less heterogeneous because all participants were from a single institution. Furthermore, because the study was cross-sectional, the long-term effects of cardiovascular symptoms could not be assessed.

## Conclusions

According to this study, hyperthyroidism was frequent in the third and fourth decades of life. Females were more likely than males to be affected. Palpitation, chest discomfort, dyspnea, and hypertension were the most prevalent cardiovascular symptoms of thyroid problems. Since cardiovascular manifestations are common in patients with thyroid disease and may be the only manifestation of thyroid disease, it is suggested that all patients with thyroid disorders be checked for cardiovascular manifestations. Also, thyroid function tests should be performed in all patients with unexplained cardiovascular disease. The findings of this study should be cautiously generalized into the broader context due to the small number of patients being studied.
